# Advanced-stage Wilms tumor arising in a horseshoe kidney of a 9-year-old child: a case report

**DOI:** 10.1186/s13256-021-03048-1

**Published:** 2021-09-15

**Authors:** Abubakar Garba Farouk, H. A. Ibrahim, A. Farate, S. Wabada, M. G. Mustapha

**Affiliations:** 1 Department of Pediatrics, Faculty of Clinical Sciences, College of Medical Sciences, University of Maiduguri, P. M. B. 1069, Maiduguri, Borno State Nigeria; 2 Department of Radiology, University of Maiduguri Teaching Hospital, P.M.B. 1414, Maiduguri, Borno State Nigeria; 3 Pediatric Surgery Unit, Department of Surgery, University of Maiduguri Teaching Hospital, P.M.B. 1414, Maiduguri, Borno State Nigeria

**Keywords:** Horseshoe kidney, Wilms tumor, Resource-poor setting, Management challenges

## Abstract

**Background:**

Horseshoe kidney (HK) is one of the most common renal fusion abnormalities, with an incidence of 1:400 in the normal population. However, Wilms tumor (WT) arising in an HK is a rare occurrence. We report the case of a 9-year-old boy who presented with an advanced WT in an HK and also highlight the management challenges in a resource-poor setting such as ours.

**Case presentation:**

The patient was a 9-year-old Nigerian boy presented to the Pediatrics Outpatient Clinic of the University of Maiduguri Teaching Hospital (UMTH) with a history of progressive abdominal swelling, weight loss, abdominal pain, and cough. Abdominal examination revealed an irregular, firm, and non-tender mass in the right lumbar region. A computed tomography (CT) scan of the abdomen showed a heterogeneously dense mass that was predominantly to the right side of the abdomen and crossed the midline to the left side, where it continued with the relatively normal renal tissue. Chest CT revealed pulmonary metastases. A diagnosis of WT in an HK was made. The patient had a 6-week course of neoadjuvant chemotherapy, and a right nephrectomy and left partial nephrectomy was performed. The final histologic diagnosis of WT was made. Radiotherapy was intended but was not available in our facility, and the parents could not afford referral to another center.

**Conclusions:**

Children with a clinically suspected HK with WT should undergo a careful imaging evaluation such as CT before any surgical intervention. Neoadjuvant chemotherapy to reduce tumor bulk might be a good treatment method to reduce surgical morbidity and aid in complete excision and potential for preserving renal function.

## Background

Wilms tumor (WT) is the most widely reported primary malignant renal tumor in childhood, with a prevalence of eight per million children [1]. WT has been associated with multiple congenital anomalies and malformations, the most common anomalies being aniridia, hemihypertrophy, and genitourinary anomalies [2, 3]. Other anatomic anomalies and syndromes associated with WT in the genitourinary tract include renal ectopia, renal hypoplasia, ureteral duplication, cryptorchidism, hypospadias, and male pseudohermaphroditism [2].

Horseshoe kidney (HK) is among the numerous renal anomalies that have been associated with WT [4]. A child with HK has a twofold increased risk of developing an embryonic tumor of the kidney compared with the general population of children without HK [5]. Even though WT is rare when it occurs in association with renal ectopia, it is generally more commonly seen with HKs than other forms of ectopia such as pelvic renal ectopia [5, 6]. There is a tendency to develop an embryonic tumor in HKs, with WT being more commonly reported than hypernephroma or renal pelvis tumor [7]. Presentation with abdominal swelling may result not only from a fulminant intra-abdominal mass, but also from fat in childhood obesity, fluid accumulation from ascites, and flatus or feces in an intestinal obstruction.

Of the 8617 patients enrolled in the National Wilms Tumor Study Group (NWTSG) over 29 years, 41 patients have been found to have WT arising in an HK, representing an incidence of 0.48%. HK was, however, not recognized preoperatively in 13 of the 41 patients despite evaluation with computed tomography (CT) [5]. Recognizing HK is difficult, but if diagnosed it warrants prophylactic surveillance utilizing frequent physical examination and/or imaging to ensure early diagnosis and treatment of WT should it occur. The surgical treatment is nephrectomy of the involved kidney and resection of the isthmus of the HK [8].

We describe a rare case of advanced WT arising from the HK of a 9-year-old child. The management challenges in a resource-poor setting such as ours are highlighted. This report also stresses the importance of screening children with genitourinary anomalies and associated disorders such as an embryonic tumor.

## Case presentation

The patient was a 9-year-old Nigerian boy living in an internally displaced person (IDP) camp with his parents, referred to the Pediatrics Outpatient Clinic of the University of Maiduguri Teaching Hospital (UMTH) from a nongovernmental organization (NGO) facility in a remote village of Borno State, northeastern Nigeria. He presented with progressive abdominal distension (more to the right side) of 5 months, abdominal pain for 2 months, and a 1-month history of cough. The patient started to experience dull-achy non-radiating abdominal pain with no relieving or aggravating factors 3 months after the swelling was noticed, and at the same time he was observed to have progressive weight loss associated with anorexia. The patient experienced an occasional global headache that he described as throbbing and fever with chills and rigors that were relieved by taking acetaminophen. There was no hematuria, facial or leg swelling, focal swelling in other parts of the body, or bone pain.

Physical examination revealed a chronically ill-looking wasted febrile child with a temperature of 38.5 °C. The patient was pale but not jaundiced. There was no significant peripheral lymphadenopathy, pedal edema, or visible varicocele. No obvious hemihypertrophy or aniridia was seen. His weight was 21 kg, which is 72% of the expected (underweight). The blood pressure was within normal limits for the patient's age, sex, and height. Abdominal examination revealed a mass in the right flank extending toward the midline. The mass was irregular, firm, non-tender, and slightly mobile. The liver and spleen were not enlarged on palpation, and the left kidney was not ballottable.

**Fig. 1:**
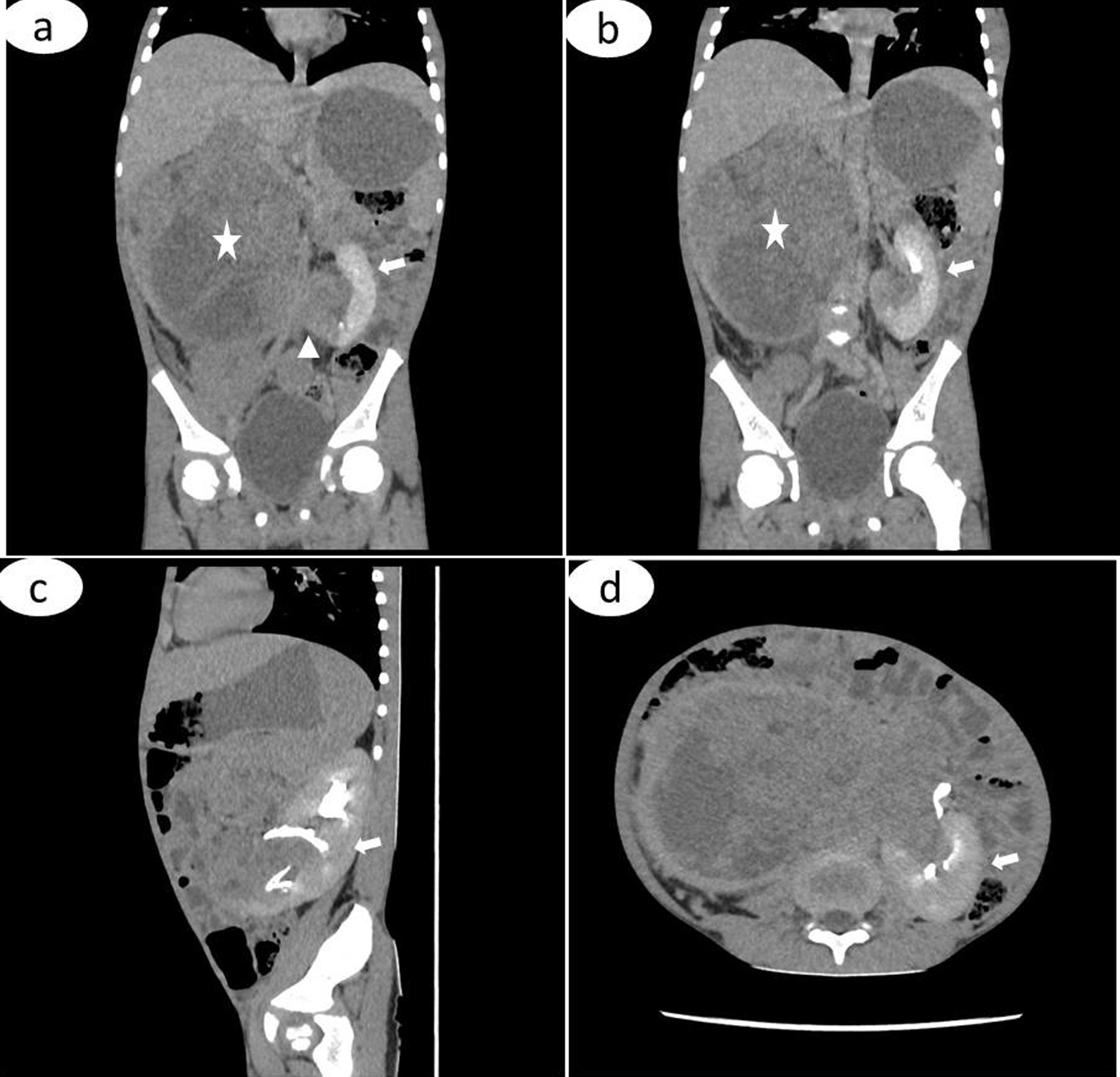
CT images; coronal contrast-enhanced images, anterior to posterior cuts (**a**, **b**) showing a heterogeneous non-enhancing low-density mass (asterisks) arising from the right kidney crossing the midline (arrow head) to involve the left kidney (arrows). There is distortion and stretching of the calyces (arrows) of the relatively spared part of the remaining left kidney (**c**, **d**)

**Fig. 2:**
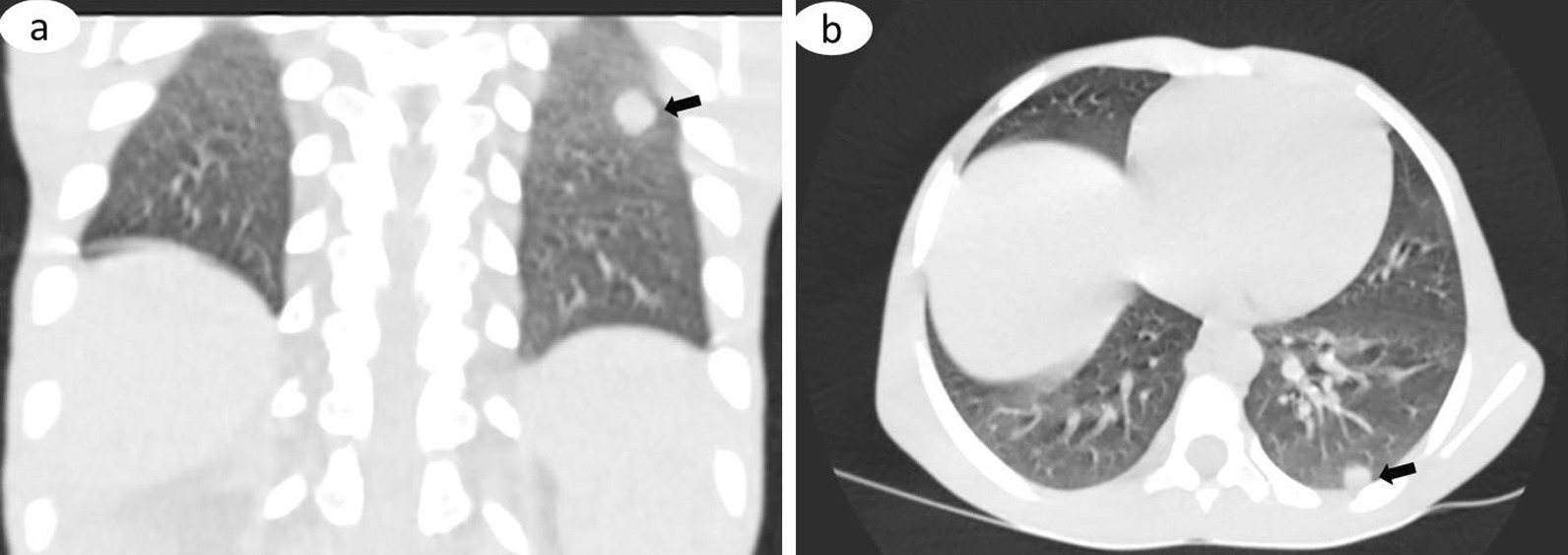
Coronal image of the chest, lung window (**a**), showing a well-defined rounded nodule in the left upper zone (arrow), and on the axial image (**b**), there is a similar nodule at the lower zone of the same left lung, abutting the posterior pleural surface (arrow). These are indicative of pulmonary metastases

An abdominal CT scan showed a heterogeneous low-density mass arising from the right kidney crossing the midline to involve the opposite left kidney, and excretory phase images revealed distortion and stretching of the calyces of the part of the left kidney that was relatively spared (Fig. 1). There were no intralesional calcifications. CT of the chest showed two discrete nodules in the left upper zone and right lower zone (Fig. 2). Urinalysis showed microscopic hematuria (3+) and ova of *Schistosoma haematobium*, but urine culture was normal. Renal and liver function tests were unremarkable.

**Fig. 3:**
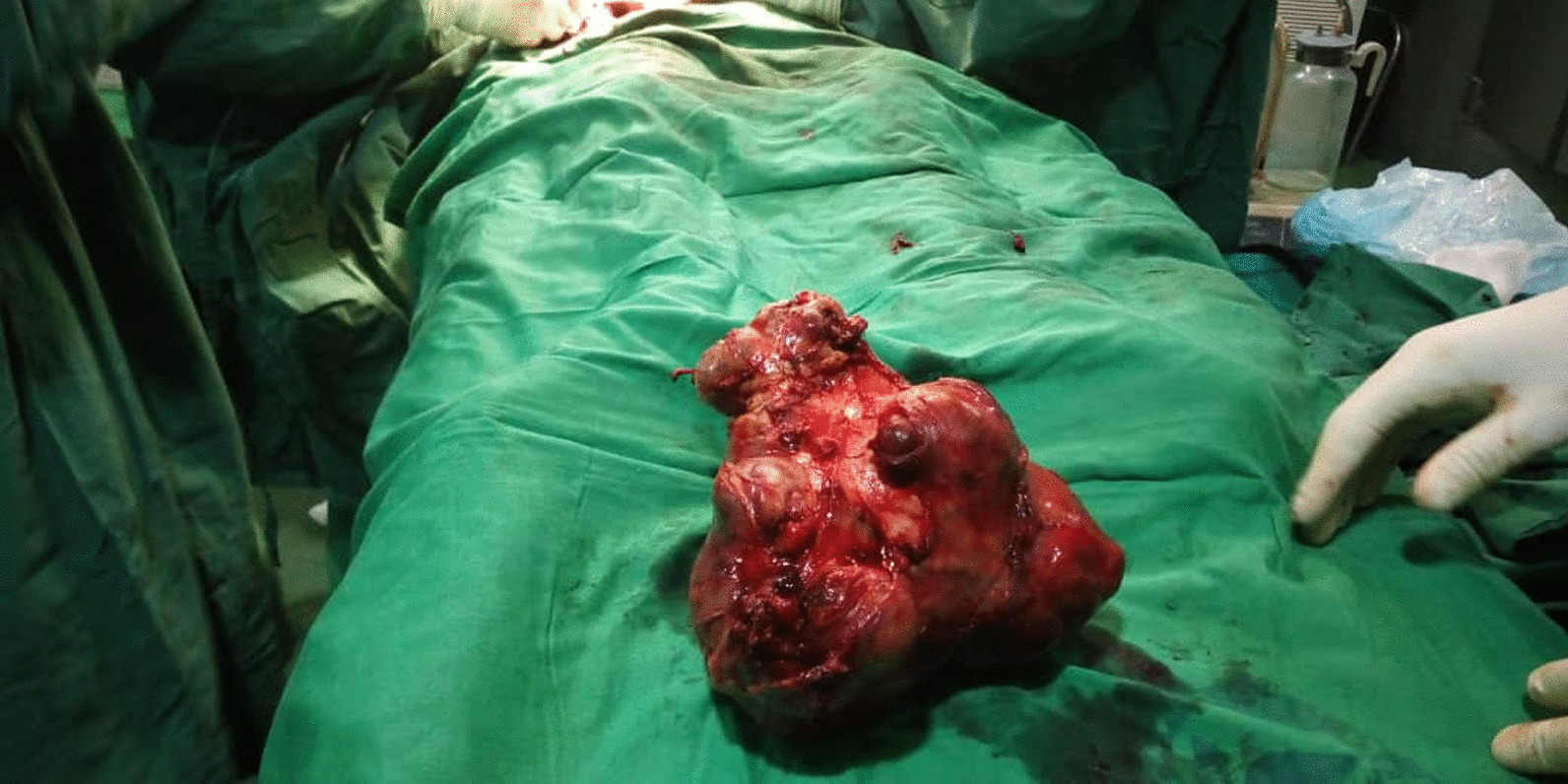
A nephrectomy specimen showing irregularity of the tumor

**Fig. 4:**
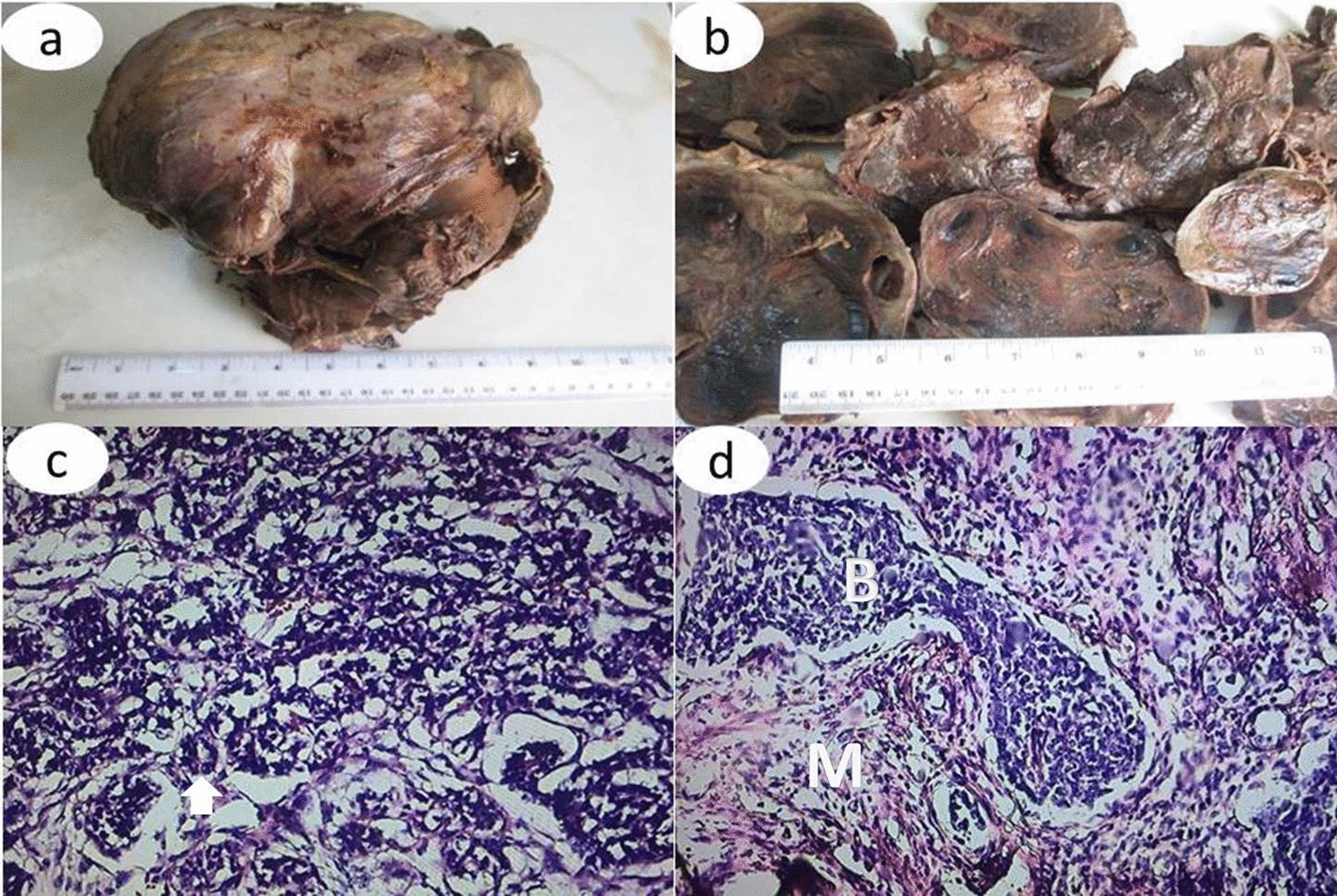
Image shows that the resected tumor (**a**) is irregular and the cut surface (**b**) appears variegated, with a mixture of tan brown, gray, and black colors. Photomicrograph show (**c**) epithelial cells (arrow) - H&E x 200; (**d**) mesenchymal cells (M) and blastemal cells (B) - H&E x 100 with high mitotic activity

A presumptive diagnosis of stage V WT in an HK was made based on the contiguous extension of the tumor from the right kidney through the isthmus to the contralateral kidney. The patient received neoadjuvant chemotherapy with vincristine and actinomycin D for 4 weeks and was subsequently scheduled for nephrectomy. The intraoperative finding was an HK with a tumor involving the right kidney, the isthmus, and the lower pole of the left kidney, confirming a preoperative diagnosis of stage V disease. Right nephrectomy, isthmusectomy, and left partial nephrectomy were performed (Fig. 3). Grossly, the resected tumor (right kidney, isthmus, and the left kidney lower pole) was irregular and firm, measuring 19 cm × 12 cm × 9 cm. The cut surface appeared variegated, with a mixture of tan brown, grey, and black colors (Fig. 4a). The histology of the tumor showed a small round blue cell composed of predominantly blastemal cells with foci of epithelial and mesenchymal elements arranged in triphasic patterns invading the renal parenchyma. There was high mitotic activity with focal areas of tumor necrosis and host cellular immune response (Fig. 4b).

The patient’s postoperative condition was unremarkable, and he was continued on adjuvant chemotherapy before he was discharged home. The desire for the patient to have radiotherapy in addition to the chemotherapy was not feasible due to the lack of a radiotherapy facility in our hospital during the period of patient management, and unfortunately, the parents could not afford referral to another hospital.

The patient was lost to follow-up after two cycles of adjuvant chemotherapy. All efforts at locating the patient were unsuccessful, as the patient together with his parents were relocated to a remote village that was not readily accessible due to the ongoing insurgency in the northeastern region of Nigeria.

## Discussion

This report demonstrates the fact that patients with a long-standing condition and diagnostic dilemma may present to a primary care facility without appropriate diagnostic tools like in our region that is devastated by insurgency, resulting in a delay in diagnosis and management. This can stress the primary care physician and the facility system, similar to the report by Devaraj [9]. HK is a rare abnormality of the kidney structure and position in the retroperitoneum. Reports on WT arising in an HK are few, with only one from the West African subregion [10]. Unlike our case, which involved bilateral WT, the case from Sokoto, Nigeria, involved a unilateral WT, and the patient died 5 days after the second course of neoadjuvant chemotherapy [10]. The two renal masses in the HK in our patient were joined at the lower poles, which is the most common finding in up to 90% of cases [11]. While the variability of vascular anatomy suggests an anomalous blood supply could be a possible cause of the abnormal position, abnormal migration of the nephrogenic cells during the early stage of gestation when the two kidneys are very close results in the fusion of the kidneys [12]. Although the etiology of WT in HK remains unknown, there is speculation that WT develops as a result of sequestration of the metanephric blastemas in the isthmus, which has the potential to undergo malignant transformation [13]. The formation of the HK likely predisposes to the occurrence of a second event which results in WT. Other renal tumors which have been described in children with HK include teratoma and rhabdomyosarcoma [14–16]. The incidence of WT arising from HK is estimated to vary from 0.4% [4] to 0.9% [17] of all WT. While most patients would have no symptoms and their HK would be discovered incidentally on imaging, our patient had rapidly progressive enlargement of the abdomen associated with pain. Wilms tumor is, however, the most common renal tumor of childhood. The survival rate of WT is over 85% in advanced countries due to early presentation and potent and effective chemotherapy and radiotherapy for such conditions [18]. Unfortunately, this remains very challenging in Nigeria and most other developing nations, largely due to poverty, delay in presentation, and lack of many treatment modalities [19].

Our patient presented with an abdominal mass and pain similar to the most symptomatic presentation of a review of 52 reported cases in the literature from 1895, when Hildebrand reported the first case of WT in an HK, to February 2004 [20]. Our patient had stage V disease at diagnosis, which is contrary to the findings of Huang *et al.*, who reviewed 52 cases, with no patient presenting with stage V disease [21]. Our patient had a tumor involving both sides of the HK and metastases to the lungs, and therefore had neoadjuvant chemotherapy followed by right nephrectomy, isthmusectomy, and left partial nephrectomy performed to help preserve renal function, and cyclic adjuvant chemotherapy with intended radiotherapy as recommended by the NWTSG or International Society of Paediatric Oncology (SIOP). However, it was not available in the center, and family care could not afford referral to the center where available.

Children with HK have an increased risk of developing WT compared with the general population. Increased routine surveillance for renal masses in children with HK is therefore justified, in similar fashion as the recommendation for children with other WT-associated syndromes such as aniridia and Beckwith–Wiedemann syndrome. Based on an incidence of 1 in 400 cases of HK, [4] the risk of having WT is extremely small and cannot justify surveillance of these patients per the NWTSG guidelines. Patients with aniridia have a 40% chance of having WT, and those with Beckwith–Wiedemann syndrome have a maximum chance of 10% [22]. The recommendations of surveillance for renal masses in patients with aniridia and Beckwith–Wiedemann syndrome include ultrasonography every 3 months until the age of 7 years, followed by physical examination every 6 months through completion of growth [23]. Children with WT arising in an HK do not differ from those with a normal kidney in terms of clinical presentation or histologic subtype [24]. HK does not unnecessarily alter the prognosis of WT; however, it can pose significant operative difficulties and postoperative problems due to anatomic variations in the shape of the HK, with a variable relationship between the great vessels and the ureters. There is usually one renal artery to each kidney. Duplicated or even triplicated renal arteries may be supplying one or both kidneys. The blood supply to the isthmus may come directly from the aorta or the renal artery, and rarely from the inferior mesenteric or the iliac arteries [24]; therefore, renal angiography might be helpful in both the diagnosis of the tumor and mapping the blood supply in WT arising in an HK for planning the surgical excision.

## Conclusions

Children with an abdominal mass mostly have delayed presentation and should, therefore, be carefully examined by imaging studies, including the use of CT. Preoperative chemotherapy in the case of WT might be a good treatment method for decreasing surgical morbidity, promoting complete excision, and preserving renal function.

However, due to delay in presentation and sometimes diagnosis, in addition to a limited number of drugs for chemotherapy and difficulties involved in surgeries of malignancies in children, increased morbidity is the rule in most developing countries like Nigeria. Other challenges include lack of facilities like radiotherapy, funds to cover the treatment of the malignancy, and other comorbid conditions. Most of these challenges have played out in the index case, as clearly depicted by his inability to return for follow-up.

## Data Availability

Not applicable.

## Abbreviations

WT: Wilms tumor
NGO: Nongovernmental organization
CT: Computed tomography
HK: Horseshoe kidney
NWTSG: National Wilms Tumor Study Group
IDP: Internally displaced person
UMTH: University of Maiduguri Teaching Hospital
SIOP: International Society of Pediatric Oncology
